# *Chlamydia trachomatis* Mimicking COVID-19 Chronic Follicular Conjunctivitis

**DOI:** 10.1155/2021/6654347

**Published:** 2021-01-30

**Authors:** Sukhmandeep Kaur, Zachary Raphael Teibel, Nada Farhat, Adam Atoot, Brett Bielory

**Affiliations:** ^1^Touro College of Osteopathic Medicine, Harlem, USA; ^2^Department of Medicine, Hackensack Meridian Health Palisades Medical Center, USA; ^3^Department of Pathology, Mount Sinai Health System, USA; ^4^Department of Medicine, Hackensack Meridian Health Palisades Medical Center, Riverside Medical Group, USA; ^5^Department of Ophthalmology, New York Eye and Ear infirmary of Mount Sinai School of Medicine, Hackensack University Medical Center, Riverside Medical Group, USA

## Abstract

**Purpose:**

To characterize the clinical presentation and report lab findings of *Chlamydia trachomatis* follicular conjunctivitis in two patients with a positive history of active COVID-19 infection. *Participants*. Two patients with follicular conjunctivitis with a recent history of COVID-19 infection.

**Design:**

Retrospective, noncomparative, case report.

**Methods:**

Demographic data including age, gender, and place of residence were recorded. A full exam with an emphasis on inflammatory characteristics and systematic workup. Sample follicles were surgically excised in selected cases, and molecular and histopathological analyses were performed.

**Results:**

Both patients were initially treated for viral conjunctivitis. After symptoms failed to resolve, biopsy results indicated that both patients were positive for chlamydia conjunctivitis and treated accordingly.

**Conclusions:**

These cases illustrate the role of biopsy as an investigative tool in chronic conjunctivitis and the importance of having a broad differential when treating patients with acute conjunctivitis.

## 1. Background

The primary causes of conjunctivitis in the United States are viral and bacterial etiologies. The primary cause of viral conjunctivitis is adenovirus. Other viral culprits include *Picornaviruses*, *Herpesvirus*, *HIV*, and even *Coronavirus* [1]. Bacterial etiologies most commonly involve Gram-positive bacteria including *Staphylococcus aureus* or *Streptococcus pneumoniae*. Gram-negative bacterial conjunctivitis is generally more severe, less common, and generally attributable to *Haemophilus influenzae* and *Moraxella Catarrhalis.*

Conjunctivitis symptom onset can be hyperacute, acute, or chronic, with 4 weeks being the cutoff for an acute classification. Patients generally present with a combination of eye redness, irritation, photophobia, blurred vision, and tearing with or without purulence. Ophthalmologists further categorize conjunctivitis as follicular or papillary depending on physical exam findings.

In Western countries, *C*. *trachomatis* infection affects 5-20% of sexually active adults. Transmission is via autoinoculation from genital secretions or eye-to-eye spread. Acute symptoms consist of unilateral or bilateral discharge and redness [2]. Patients present with preauricular lymphadenopathy, prominent follicles in the inferior fornix, or upper tarsal conjunctiva [3]. If left untreated, acute chlamydial conjunctivitis can become chronic, with scarring of the conjunctiva, xerosis, and entropion trichiasis [4]. Chlamydial conjunctivitis can be distinguished from other causes with nucleic acid amplification tests such as PCR or Giemsa staining of tarsal conjunctival scrapings [5].

Coronavirus conjunctivitis can be unilateral or bilateral and generally causes chemosis, watery discharge with or without systemic symptoms [6]. While *Adenovirus* is more common, the classification of COVID-19 as a pandemic by the World Health Organization (WHO) means it must be ruled out via RT-PCR and antibody testing before exploring other pathogens [7–9]. COVID-19-infected patients were found to contain Coronavirus RNA in their tears and conjunctival secretions [10, 11].

A study in China previously reported conjunctivitis in 0.8% COVID-19 patients (9 of 1099) [10]. COVID-19 penetrates into the conjunctiva the same way it does the lungs, using angiotensin-converting enzyme subtype 2 receptor on corneal epithelial cells via the assistance of heparan sulfate proteoglycan receptors [12]. If the condition persists without treatment, major complications such as punctate keratitis with subepithelial infiltrates, bacterial superinfection, conjunctival scarring, and symblepharon, severe dry eye, irregular astigmatism, corneal ulceration with persistent keratoconjunctivitis, corneal scarring, and chronic infection can occur [13]. Treatment includes supportive therapy such as cold compress, artificial tears, and antihistamines until the viral infection subsides.

## 2. Case Presentation

### 2.1. Case #1

A 25-year-old male presented with a 2-month history of bilateral eye redness, irritation, and tearing. One month prior to presentation, he was treated with a course of moxifloxacin eye drops but symptoms recurred 2 days thereafter. He had been diagnosed with COVID-19 using a nasopharyngeal specimen collected and analyzed via RT-PCR (LabCorp Inc., Raritan, NJ) in March 2020.

On exam, the patient had normal vision and intraocular pressure in both eyes and one 1.0 mm follicle on the right inferior tarsal of the right eye (OD) without discharge or injection. The left eye (OS) had one 1.5 mm follicle on the left inferior tarsal and ptosis of the left upper eyelid. There was bilateral bulbar and palpebral conjunctival injection.

Past medical history included a history of LASIK and genital herpes controlled with oral valacyclovir.

### 2.2. Case #2

A 19-year-old male presented with 1-month history of irritation and tearing in the left eye (OS) and redness in bilateral eyes (OU). The patient was initially treated with ciprofloxacin eye drops and erythromycin ointment, but his condition continued to worsen. He had been diagnosed with COVID-19 using a nasopharyngeal specimen collected and analyzed via RT-PCR. He had been diagnosed with COVID-19 via RT-PCR (Abbott Laboratories Inc., Lake Forest, IL) July 2020.

**Figure 1:**
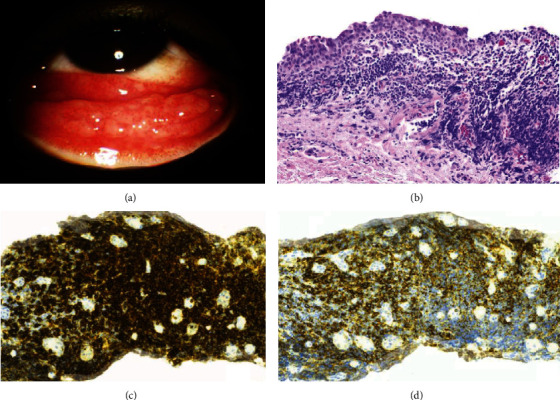
Clinical findings show prominent follicular reaction with conjunctival mucosa (a). Microscopically, a mixed chronic inflammatory infiltrate is seen within the subepithelium (b: H&E, 20x). Immunohistochemical studies highlight a mixture of B cells (c: CD20, 20x) and T cells (d: CD3; 20x).

On exam, visual acuity was 20/60 bilaterally with normal intraocular pressure and bilateral redness of the bulbar conjunctiva. A prominent follicular reaction of the lower conjunctiva was seen, and superficial episcleral injection in both eyes without pain was noted (Figure 1).

Both patients had conjunctival biopsy and were tested for coinfections of chlamydia, HSV, adenovirus, and gonorrhea (Table 1). Biopsy of the left eye revealed benign epithelium with subepithelial tissue showing reactive lymphoid hyperplasia highlighted with B and T cell immunostains CD20 and CD3, respectively.

## 3. Diagnostics

### 3.1. Treatment

Both patients' symptoms resolved with a course of oral doxycycline and erythromycin.

### 3.2. Outcome and Follow-Up

Both patients recovered without recurrence or lasting visual complications. Of note, case 2 also had a biopsy-positive diagnosis of HSV and was started on a course of acyclovir.

## 4. Discussion

Viral and bacterial conjunctivitis often presents with many overlapping symptoms. Patients have redness, chemosis, and tearing. Physicians may be likely to attribute acute conjunctivitis to COVID-19 in patients with this as a preexisting diagnosis. However, like COVID-19, chlamydial conjunctivitis can also be spread via eye secretion and can also progress to a chronic form. Although supportive treatment is the current standard for COVID-19 conjunctivitis, chlamydia necessitates the use of oral antibiotics and, without proper treatment, it can cause conjunctival scarring, xerosis, and entropion trichiasis. As such, physicians must be cognizant of maintaining a broad differential for acute conjunctivitis in the post-COVID world [14].

## 5. Learning Points

1. Consider tissue diagnosis via conjunctival biopsy when empiric therapy fails to resolve cases of acute or chronic conjunctivitis
2. Secondary causes of conjunctivitis must be treated systemically along with assessing it from an ocular standpoint

## Figures and Tables

**Table 1:** Patient-specific data that depicts coinfection of COVID-19 and chlamydia.

Pathogen tested	COVID-19	Chlamydia	HSV PCR	Adenovirus	Gonorrhea PCR
Case #1	Positive 3/2020	Positive 6/2020	Negative	Negative	Negative
Case #2	Positive 7/2020	Positive 8/2020	Positive 8/2020	Negative	Negative

## Data Availability

The literature review data used to support the findings of this case report are included within the article.
